# LINC01089 blocks malignant progression of thyroid cancer by binding miR-27b-3p to enhance the FBLN5 protein level

**DOI:** 10.1007/s12672-022-00580-4

**Published:** 2022-10-28

**Authors:** Yong-qin Pan, Kun-song Huang, Tsz-Hong Chong, Jin-yi Li

**Affiliations:** Department of Thyroid Surgery, The First Affiliated Hospital of Jinan University, Guangzhou, 510632 China

**Keywords:** lncRNA, Metastasis, microRNA, Thyroid cancer

## Abstract

LINC01089 suppresses the malignant progression of breast, colorectal, and non-small cell lung cancers. However, the function of LINC01089 in thyroid cancer has not yet been elucidated. Here, The Cancer Genome Atlas (TCGA) database showed that LINC01089 expression is remarkably reduced in thyroid cancer tissues. Lower LINC01089 expression was correlated with higher tumor stage and regional lymph node metastasis. Furthermore, LINC01089 overexpression effectively blocked thyroid cancer cell proliferation, migration, and invasion. LINC01089 acted as a competing endogenous RNA for miR-27b-3p, thus inhibiting miR-27b-3p expression. miR-27b-3p overexpression promoted the proliferation, migration, and invasion of thyroid cancer, reversing the effect of LINC01089 overexpression on thyroid cancer. Fibulin-5 (FBLN5) was discovered as a target of miR-27b-3p in thyroid cancer. FBLN5 expression was found to be underexpressed in thyroid cancer and was enhanced and reduced by LINC00987 overexpression and miR-27b-3p overexpression, respectively. Furthermore, FBLN5 knockdown promoted the malignant progression of thyroid cancer cells by counteracting the effect of LINC00987. In conclusion, LINC01089 plays a tumor-suppressive role by binding miR-27b-3p to increase FBLN5 expression, confirming that LINC01089 has tremendous potential to become a therapeutic target for thyroid cancer treatment.

## Introduction

Thyroid cancer, a notable endocrine cancer, is the fourth most prevalent cancer worldwide, and its morbidity rate has rapidly increased over the past few decades [1]. Patients with thyroid cancer have a satisfactory prognosis after treatment, with a 5-year survival rate of over 98% [2]. Nevertheless, thyroid cancer has a strong tendency to metastasize to neck lymph nodes (20–50%) and exhibit distant metastasis (3–15%), which promotes tumor recurrence and results in a 5-year survival rate of metastatic patients as low as ~ 50% [3–6]. Therefore, identifying novel therapeutic targets for use in the treatment of metastatic thyroid cancer is critical.

Long noncoding RNAs (lncRNAs) play an important role in regulating thyroid cancer initiation and progression. MALAT1 and OIP5-AS1, which contribute to thyroid cancer growth and progression, play an oncogenic role [7, 8]. In contrast, SLC26A4-AS1 and PTCSC3, which alleviate thyroid cancer initiation and progression, have tumor-suppressive effects [9, 10]. Therefore, further understanding of the role of lncRNAs in thyroid cancer progression is crucial. As reported, LINC01089 suppresses the malignant progression of non-small cell lung cancer (NSCLC), breast cancer, and colorectal cancers [11–13]. Whereas, LINC01089 knockdown enhanced chemosensitivity for sorafenib in hepatocellular carcinoma cells [14]. However, the function of LINC01089 in thyroid cancer has not yet been elucidated.

Notably, many lncRNAs that ‘sponge’ specific miRNAs via ceRNAs function to control tumor development. LINC01089 can sponge miR-27a/27b/3187-3p in breast, gastric, and NSCLC cancers, respectively [12, 13, 15]. miRNAs play an important role in the regulation of thyroid cancer. miRNA such as miR-144-3p reduced the malignant progression of thyroid cancer while let-7e-5p promoted the malignant progression of thyroid cancer [16, 17]. Therefore, we hypothesized that miRNAs might be sponged by LINC01089, which prevents the progression of thyroid cancer. Fibrin enhances cell-to-cell adhesion in the cellular microenvironment and also affects cell behavior [18]. Fibulin-5 (FBLN5), a fibrin, has been shown to enhance endothelial cell adhesion [19]. FBLN5 reduced the invasion, mammospheres formation, and proliferation capacity of cancer cells via silencing β-catenin phosphorylation in breast cancer and NSCLC, acting as a cancer suppressor gene [20–22]. Whether FBLN5 acts downstream of LINC01089 regulation in thyroid cancer is unclear.

Here, the key functions and mechanisms of LINC01089 in the malignant progression of thyroid cancer were studied. The results demonstrate the potential of LINC01089 for use as a therapeutic target in the treatment of thyroid cancer.

## Materials and methods

### Bioinformatic analyses

LINC01089 expression and differential low expression of mRNA in thyroid cancer and normal thyroid samples were analyzed using GEPIA2.0. The possible adsorbed miRNA of LINC01089 was analyzed via starBase 3.0 [23] and LncBase Predicted v.2. Differentially expressed miRNAs in thyroid cancer were analyzed using OncomiR [24]. MiR-27b-3p and miR-129-5p expression were found in thyroid cancer using starBase 3.0 [23]. The subcellular localization of LINC01089 expression was determined using the lncLocator (http://www.csbio.sjtu.edu.cn/bioinf/lncLocator/).

### Cell culture and transfection

Cells from a normal thyroid follicular epithelial cell line (Nthy-ori 3-1) and two thyroid cancer cell lines (TPC-1 and CAL-62) (ZQXZ, Shanghai, China) were cultured according to the recommendations. The LINC01089 sequence (NR_152740.1) was compounded and cloned into pcDNA3.1 (Promega, Madison, WI, USA) and named ovLINC01089. Empty pcDNA3.1 acted as a negative control (ovNC). Additionally, NC mimic, miR-27b-3p mimic (miR mimic), siRNA for FBLN5 (siFBLN5), and NC siRNA (siNC) were procured from RiboBio (Guangzhou, China). Lipofectamine 3000 (Invitrogen, Carlsbad, CA, USA) was used for transfecting experiments.

### Reverse-transcription quantitative polymerase chain reaction (RT-qPCR)

Total RNA was extracted from Nthy-ori 3-1, TPC-1, and CAL-62 cells using TRIzol reagent (Invitrogen). The quality and concentration of RNA were detected using an Eppendorf BioPhotometer Plus (Munchen, Germany). RNA was reverse-transcribed using the RT Master Mix Kit (Promega, Madison, WI, USA), and qPCR was performed using SYBR Green qPCR SuperMix (Invitrogen) with the ABI PRISM^®^ 7500 Sequence Detection System (Invitrogen). The primer sequences were as follows: LINC01089: 5′-CAGCGCTCAGCCTTCAGTAA-3′ (forward) and 5′-CGTTTATTGAGAGGCAGTTGTG-3′ (reverse); β-actin: 5′-GCATGGGTCAGAAGGATTCCT-3′ (forward) and 5′-TCGTCCCAGTTGGTGACGAT-3′ (reverse); miR-27b-3p: 5′-ACACTCCAGCTGGGTTCACAGTGGCTAAGTT-3′ (forward) and 5′-CTCAACTGGTGTCGTGGA-3′ (reverse); and U6: 5′-CTCGCTTCGGCAGCACA-3′ (forward) and 5′-AACGCTTCACGAATTTGCGT-3′ (reverse). LINC01089 and miR-27b-3p expression were normalized to GAPDH and U6, respectively.

### Cell proliferation, migration, and invasion assays

The proliferation of TPC-1 and CAL-62 cells was assessed at 24, 48, and 72 h using the Cell Counting Kit-8 assay (CCK8; Dojindo, Kumamoto, Japan) at 450 nm. Migration and invasion of TPC-1 and CAL-62 cells were analyzed using the Transwell assay with or without Matrigel (BD Biosciences), based on a previous study [13].

### Dual-luciferase reporter assay

The wild-type and mutational-type sequences of LINC01089 and FBLN5 3′-UTR sequences were compounded and cloned into the pmirGLO luciferase reporter vector (Promega). Luciferase reporter vectors and miR-27b-3p mimics were co-transfected into HEK-293T cells. After transfection for 24 h, luciferase activity was analyzed using the Dual-Luciferase kit (Promega) and was normalized to Renilla luciferase activity.

### Western blot

FBLN5 was analyzed by western blotting based on a previous study [13]. Briefly, the total protein (30 μg per lane) was isolated from Nthy-ori 3-1, TPC-1, and CAL-62 cells using 10% SDS-PAGE, and then the proteins were transferred onto methanol-pretreated polyvinylidene fluoride membranes. Next, membrane blocking and primary rabbit monoclonal antibody treatment were performed using the following antibodies: anti-fibulin 5 (1:3000, ab109428, Abcam) and anti-GAPDH (1:10,000, ab181602, Abcam), respectively. A dilution of Goat Anti-Rabbit IgG H&L (HRP) (1:10,000, ab205718, Abcam) was added. Enhanced chemiluminescent reagent (Thermo Scientific Pierce, Rockford, IL, USA) was used to visualize the protein abundance.

### Argonaute2 (AGO2)-RNA immunoprecipitation (RIP) assays

The AGO2-RIP assay was performed using the RIP Kit (Boxin, Guangzhou, China) following the manufacturer’s instructions. Then, miR-27b-3p and LINC01089 expressions were analyzed by RT-qPCR.

### Statistical analyses

The statistical analyses were performed using SPSS 21.0 (IBM SPSS Statistics). Experimental data that had consistent normal distributions are shown as the mean ± standard deviation (SD). One-way analysis of variance (ANOVA) was used to study the differences between three groups, followed by Tukey’s post hoc test to study the differences between two groups. Differences between the two groups were assessed using the unpaired t-test. p < 0.05 was considered statistically significant.

## Results

### LINC01089 downregulation was related to higher tumor size and the metastasis of regional lymph nodes in thyroid cancer

**Fig. 1:**
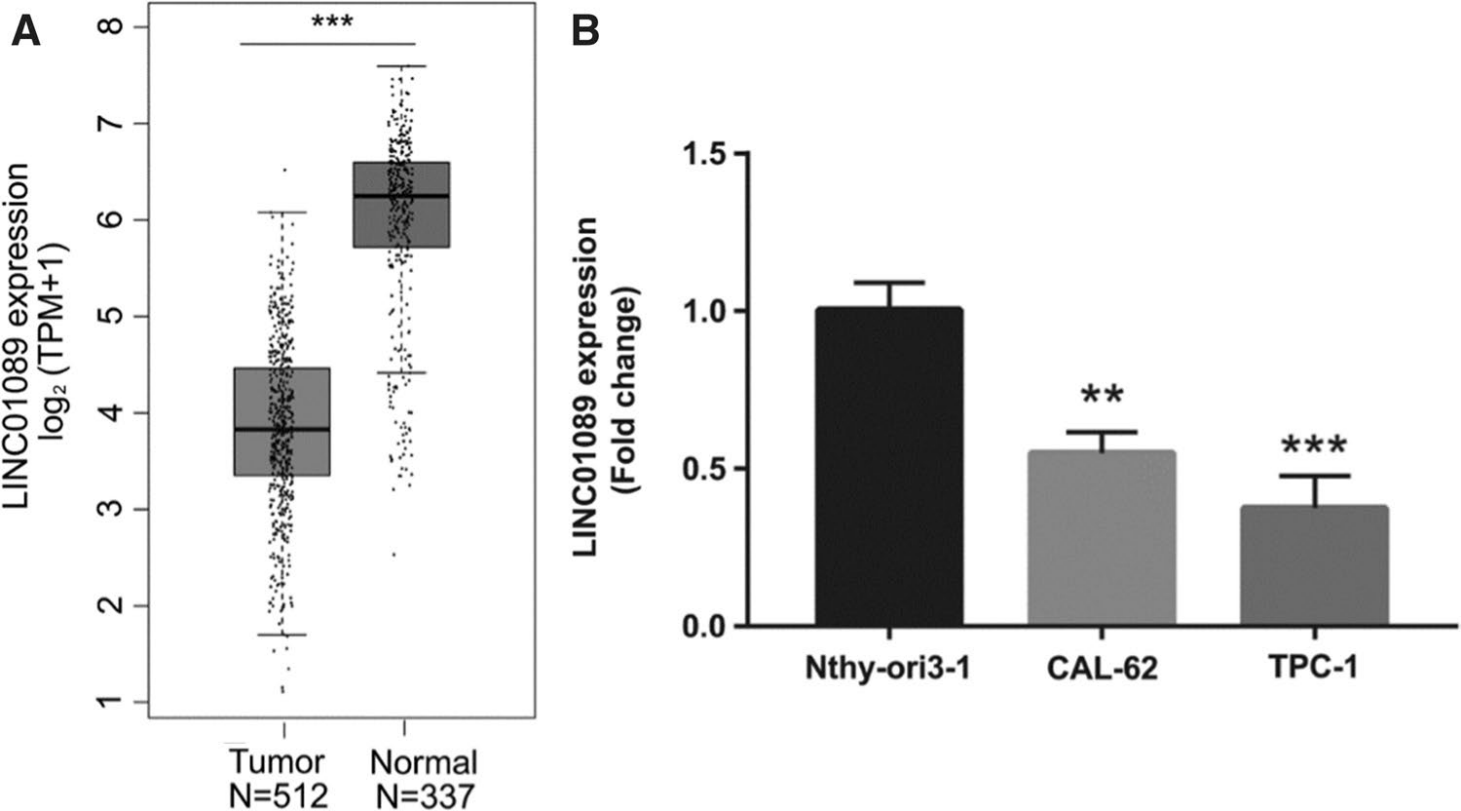
LINC01089 expression was low in thyroid cancer tissues and cells. **A** LINC01089 expression was low in thyroid cancer tissues, as determined by GEPIA analysis. **B** LINC01089 expression in normal Nthy-ori 3-1 cells and thyroid cancer cells (TPC-1 and CAL-62) was measured by RT-qPCR

First, the LINC01089 expression was analyzed using GEPIA. Figure 1A shows that LINC01089 expression was lower in thyroid cancer samples compared with that in normal samples. Moreover, LINC01089 expression was lower in thyroid cancer cells (TPC-1 and CAL-62) compared with that in normal Nthy-ori 3-1 cells (Fig. 1B). Additionally, lower LINC01089 expression was related to female patients, greater tumor size, and the metastasis of regional lymph nodes (Table 1). LINC01089 was not significantly associated with age, distant metastasis stage, pathologic stage, progression-free interval, and overall survival in patients with thyroid cancer (Table 1). The results suggested that low expression of LINC01089 may be associated with tumor proliferation and migration.

**Table 1:** The relationship between LINC01089 and the clinical characteristics

Characteristic	Low expression	High expression	p
n	255	255	
Age, meidan	46 (35, 61)	46 (34, 56)	0.340
Gender, n (%)			0.047
Female	196 (38.4%)	175 (34.3%)	
Male	59 (11.6%)	80 (15.7%)	
T stage, n (%)			0.004
T1	62 (12.2%)	81 (15.9%)	
T2	73 (14.4%)	94 (18.5%)	
T3	104 (20.5%)	71 (14%)	
T4	15 (3%)	8 (1.6%)	
N stage, n (%)			0.025
N0	101 (22%)	128 (27.8%)	
N1	127 (27.6%)	104 (22.6%)	
M stage, n (%)			0.506
M0	137 (46.4%)	149 (50.5%)	
M1	3 (1%)	6 (2%)	
Pathologic stage, n (%)			0.761
Stage I	139 (27.4%)	147 (28.9%)	
Stage II	25 (4.9%)	27 (5.3%)	
Stage III	61 (12%)	52 (10.2%)	
Stage IV	30 (5.9%)	27 (5.3%)	
OS event, n (%)			0.446
Alive	245 (48%)	249 (48.8%)	
Dead	10 (2%)	6 (1.2%)	
PFI event, n (%)			0.314
Alive	224 (43.9%)	232 (45.5%)	
Dead	31 (6.1%)	23 (4.5%)	

T: Tumor size; N: the metastasis of regional lymphnodes; M: distant metastasis; OS: overall survival; PFI: progression-free interval. T1: maximum diameter of tumor ≤ 2 cm and confined to the thyroid; T2: maximum diameter of tumor > 2 cm but ≤ 4 cm and confined to the thyroid; T3: maximum diameter of tumor > 4 cm and confined to the thyroid gland, or tumor of any size that invades the band muscle outside the thyroid gland under the naked eye; T4: includes gross extracorporeal invasion of the thyroid gland. N0: No regional lymphnodes metastasis; N1: with regional lymphnodes metastasis. M0: No distant metastases; M1: with distant metastasis

### LINC01089 inhibits cell proliferation and migration in thyroid cancer

**Fig. 2:**
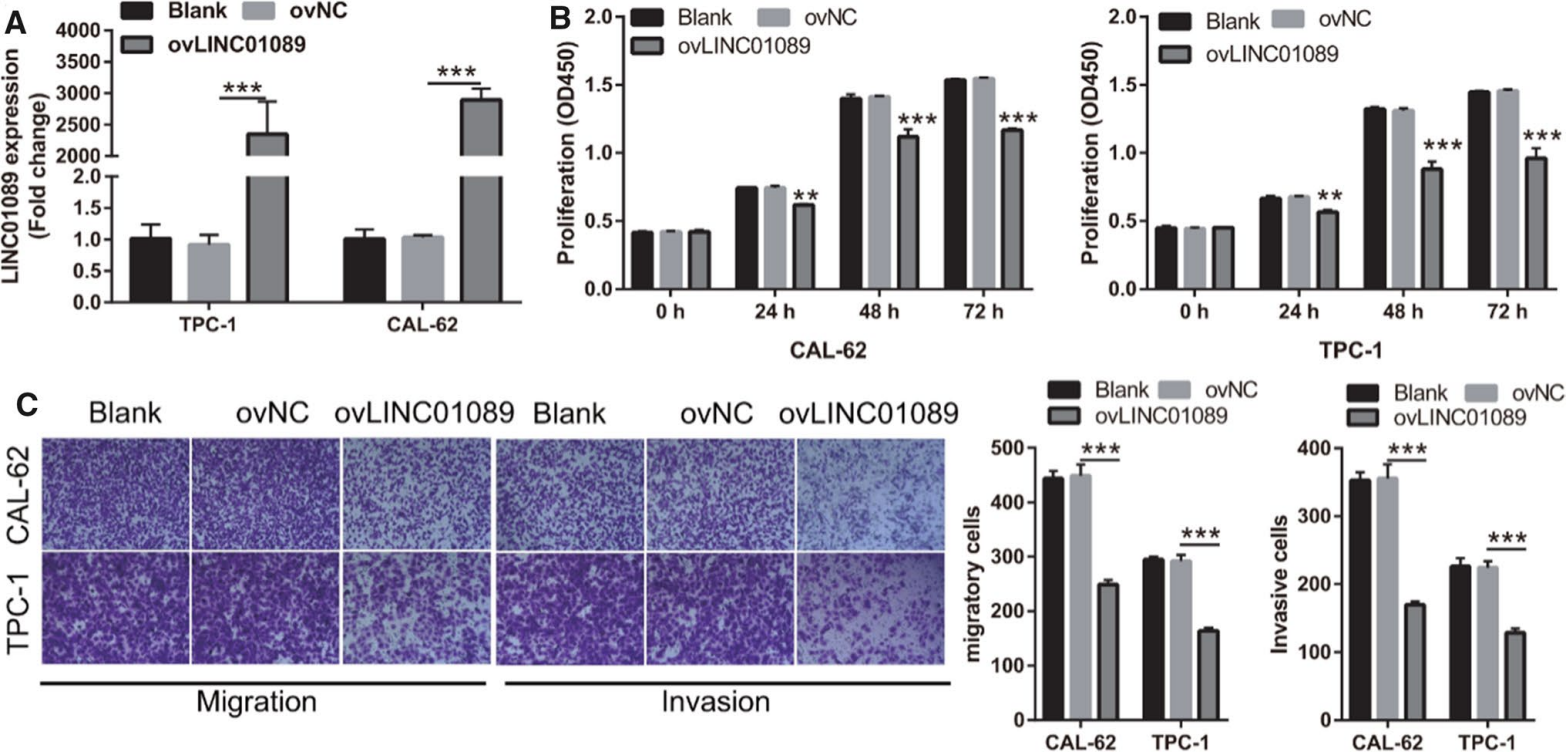
LINC01089 inhibits proliferation, migration, and invasion capacity of thyroid cancer. **A** LINC01089 expression in blank, ovNC-transfected, and ovLINC01089-transfected TPC-1 and CAL-62 cells was measured by RT-qPCR at 48 h after transfection. **B** The proliferation of TPC-1 and CAL-62 cells was evaluated by CCK-8 assay at 48 h after transfection. **C** Migration and invasion capacity of TPC-1 and CAL-62 cells were assessed by Transwell assay at 48 h after transfection (magnification, 100×). Histograms represent the mean ± SD (n = 3). **p < 0.01, ***p < 0.001

To validate the effect of LINC01089, we enhanced LINC01089 expression by transfecting pcDNA3.1/LINC01089. LINC01089 expression was higher in the ovLINC01089 group compared with that in the ovNC group in both TPC-1 and CAL-62 cells (Fig. 2A). Furthermore, the proliferation, migration, and invasion capacity of TPC-1 and CAL-62 cells were decreased in the ovLINC01089 group compared with that in the ovNC group (Fig. 2B and C).

### LINC01089 functions as a ceRNA for miR-27b-3p in thyroid cancer

**Fig. 3:**
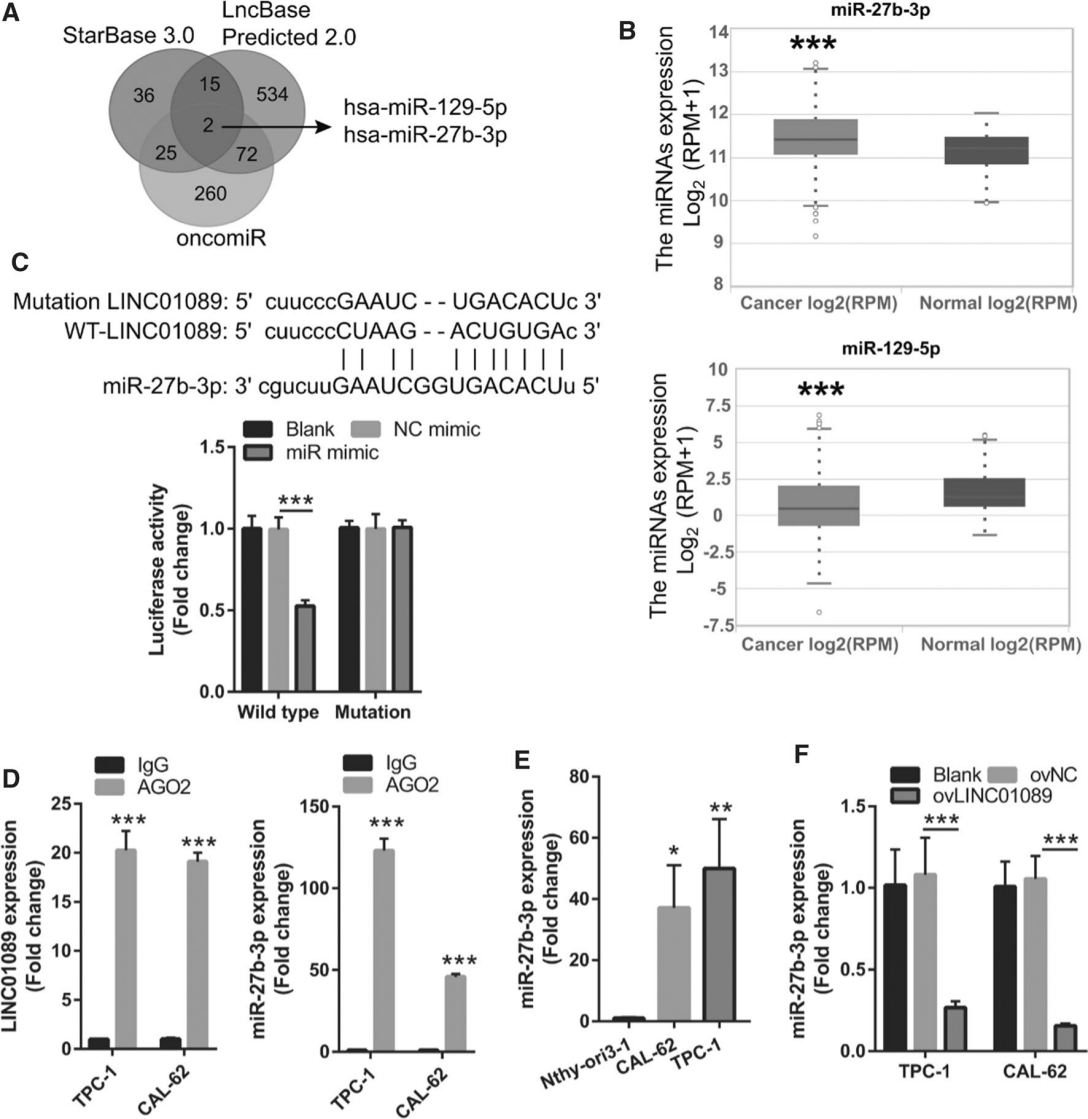
LINC01089 could sponge and inhibit miR-27b-3p expression in thyroid cancer. **A** Three circles represent the targeted miRNAs of LINC01089 (starBase 3.0 and LncBase Predicted 2.0 websites) and differential expression of miRNAs in thyroid cancer (oncomiR). The middle part represents the intersection of miRNAs. **B** Differential expression of miR-27b-3p and miR-129-5p via TCGA data analysis. **C** The binding of LINC01089 to miR-27b-3p was predicted via the starBase 3.0 website. The binding of LINC01089 to miR-27b-3p was confirmed via dual-luciferase reporter gene assay. **D** miR-27b-3p and LINC01089 expressions were analyzed by RT-qPCR after AGO2-RIP analysis. **E** miR-27b-3p expression in thyroid cancer cells (TPC-1 and CAL-62) and normal Nthy-ori 3-1 cells was measured via RT-qPCR. **F** miR-27b-3p expression in blank, ovNC-transfected, and ovLINC01089-transfected TPC-1 and CAL-62 cells was analyzed by RT-qPCR at 48 h after transfection. Histograms represent the mean ± SD (n = 3). *p < 0.05, **p < 0.01, ***p < 0.001

LINC01089 expression mainly occurs in the cytosol according to lncLocator analysis, suggesting that LINC01089 may play a regulatory role by adsorbing miRNA. The predicted targets of LINC01089 were analyzed using starBase 3.0 and LncBase Predicted v.2. In addition, differentially expressed miRNAs in thyroid cancer were analyzed using OncomiR. The overlapping part of the three sets of data revealed two potential target miRNAs (Fig. 3A). Among the two miRNAs, miR-27b-3p expression was markedly overexpressed, while miR-129-5p expression was markedly reduced in the tumor samples from TCGA data (Fig. 3B). LINC01089 had binding sites with miR-27b-3p (Fig. 3C). The luciferase activity was markedly lowered in the miR-27b-3p mimic + wild-type-LINC01089 group, while there was no marked effect in the miR-27b-3p mimic + mutant-LINC01089 group, compared with the corresponding NC mimic + group (Fig. 3C). The AGO2-RIP analysis results showed that LINC01089 and miR-27b-3p expression in the AGO2 group were markedly higher than those in the IgG group (Fig. 3D). AGO2-RIP and luciferase activity analysis indicated that LINC01089 could sponge to miR-27b-3p. Moreover, miR-27b-3p expression in TPC-1 and CAL-62 cells was significantly higher than that in Nthy-ori 3-1 cells (Fig. 3E). Intriguingly, RT-qPCR analysis showed a decline of miR-27b-3p expression in TPC-1 and CAL-62 cells transfected with ovLINC01089 (Fig. 3F). These findings suggest that LINC01089 can sponge and inhibit miR-27b-3p expression in TPC-1 and CAL-62 cells.

### miR-27b-3p overexpression weakened the LINC01089 effect in thyroid cancer

**Fig. 4:**
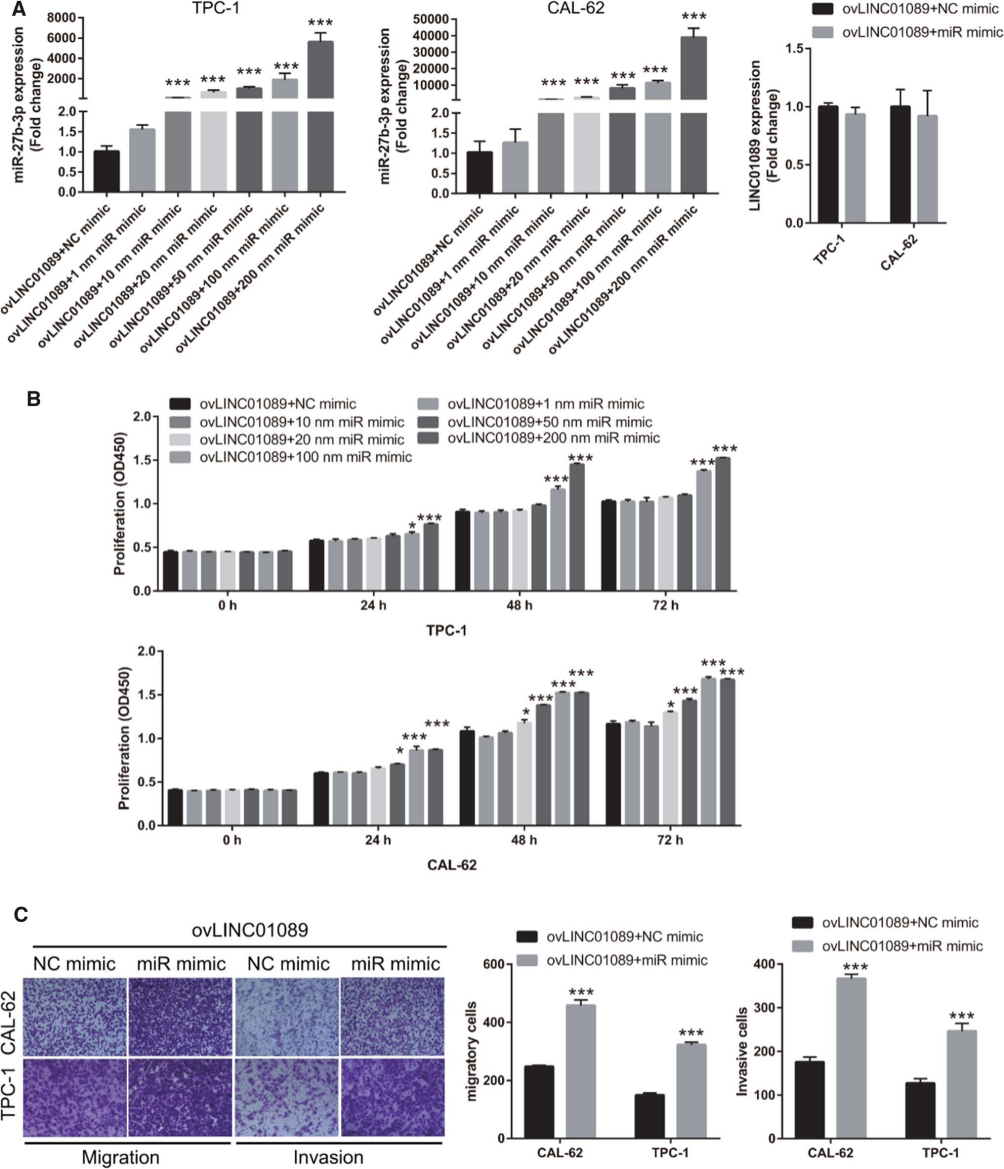
miR-27b-3p overexpression promoted the malignant progression potential of ovLINC01089-transfected thyroid cancer cells. **A** miR-27b-3p expression in TPC-1 and CAL-62 cells was measured via RT-qPCR after co-transfection with ovLINC01089 and the different concentrations of miR (miR-27b-3p) mimic. LINC01089 expression in TPC-1 and CAL-62 cells was measured via RT-qPCR after co-transfection with ovLINC01089 and 200 nM miR mimic. **B** Proliferation was evaluated by the CCK-8 assay after co-transfection ovLINC01089 and the different concentrations of miR mimic. **C** Migration and invasion were assessed by the Transwell assay after co-transfection ovLINC01089 and 200 nM miR mimic (magnification, 100×). Histograms represent the mean ± SD (n = 3). *p < 0.05 and ***p < 0.001

Next, we investigated whether miR-27b-3p participates in the effect of LINC01089 on the biological function of thyroid cancer. First, to assess whether LINC01089 would saturate with the increase of miR-27b-3p, we performed rescue experiments and co-transfected ovLINC01089 and the different concentrations of miR-27b-3p-mimic into TPC-1 and CAL-62 cells. Compared with that in the ovLINC01089 + NC mimic group, miR-27b-3p expression in the ovLINC01089 + 10/20/50/100/200 nM miR mimic groups was high in both TPC-1 and CAL-62 cells (Fig. 4A). Whereas, miR-27b-3p expression exhibited no change between ovLINC01089 + NC mimic and ovLINC01089 + 1 nM miR mimic groups (Fig. 4A). In addition, LINC01089 expression exhibited no change between ovLINC01089 + NC mimic and ovLINC01089 + miR mimic (200 nM) groups, suggesting that miR-27b-3p overexpression did not affect LINC01089 expression (Fig. 4A). Meanwhile, compared with that in the ovLINC01089 + NC mimic group, the proliferation capacity of thyroid cancer cells was significantly increased in the ovLINC01089 + 100/200 nM miR mimic group in TPC-1 cell and in the ovLINC01089 + 20/50/100/200 nM miR mimic group in CAL-62 cell, while other ovLINC01089 + miR mimic groups had no significant change (Fig. 4B). According to the expression results of miR-27b-5p and proliferation results in TPC-1 and CAL-62 cells (Fig. 4A and B), it showed that only when the expression of miR-27b-5p reached a certain level, the effect of LINC01089 on cell proliferation could be significantly reversed. This result shows that LINC01089 will saturate with the increase of miR-27b-3p, causing miR-27b-3p to reverse LINC01089 effect. Compared with that in the ovLINC01089 + NC mimic group, the migration and invasion capacity of thyroid cancer cells were significantly increased in the ovLINC01089 + miR mimic (200 nM) group (Fig. 4C). This result suggested that miR-27b-3p overexpression promotes the malignant progression potential of thyroid cancer cells and weakens the LINC01089 effect.

### FBLN5 is a possible target of LINC01089/miR-27b-3p

**Fig. 5:**
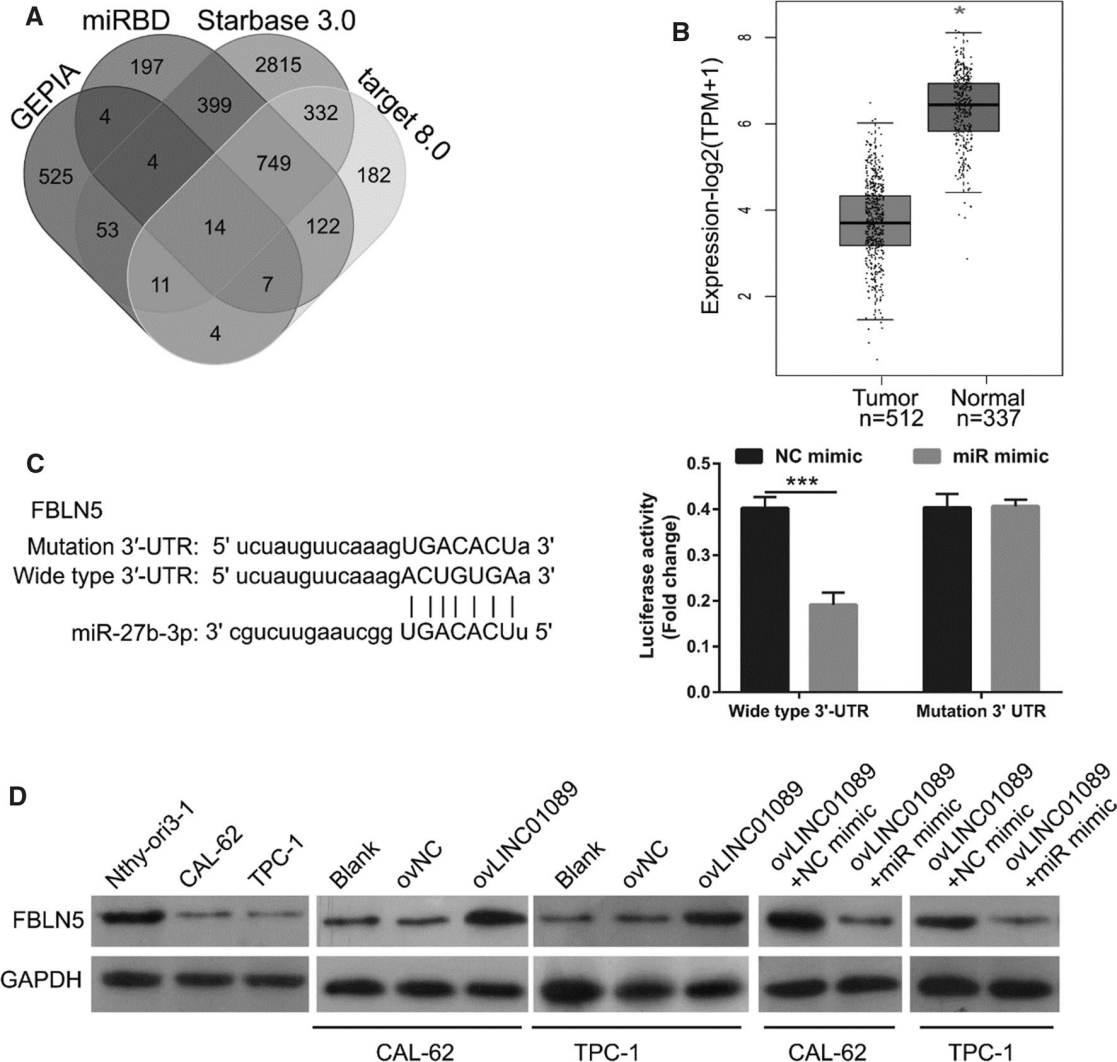
FBLN5 was the downstream target of LINC01089/miR-27b-3p. **A** Significantly decreased genes in thyroid cancer were analyzed by GEPIA. Then, the possible target of miR-27b-3p was analyzed by miRDB, Starbase 3.0, and targetscan 8.0. The intersection of the above two sets of results indicated 14 potential target genes. **B** FBLN5 mRNA expression in thyroid cancer was determined by GEPIA analysis. **C** The binding between miR-27b-3p and FBLN5 3ʹ-UTR was analyzed by the luciferase assay. **D** FBLN5 protein in transfected TPC-1 and CAL-62 cells was analyzed by western blotting at 48 h after transfection

First, significantly decreased genes in thyroid cancer were analyzed by GEPIA. Then, the possible target of miR-27b-3p was analyzed via Starbase 3.0, miRDB, and targetscan 8.0. The intersection of the above two sets of results indicated 14 potential target genes (Fig. 5A). Among the 14 potential target genes, FBLN5 mRNA expression was significantly decreased in thyroid cancer, as determined by GEPIA analysis (Fig. 5B). The luciferase assay showed that after co-transfection with wild-type FBLN5 3ʹ-UTR, the relative fluorescence value in the miR-27b-3p mimic group was significantly lower than that of the NC group, while the relative fluorescence value showed no significant change between the miR-27b-3p-mimic + mutant FBLN5 3ʹ-UTR and NC-mimic + mutant FBLN5 3ʹ-UTR groups (Fig. 5C), suggested that FBLN5 3ʹ-UTR is bonded with miR-27b-3p. In addition, the FBLN5 protein level was significantly decreased in both thyroid cancer cells. The FBLN5 protein level was increased after LINC01089 overexpression, while it was reduced in TPC-1 and CAL-62 cells after ovLINC01089 and miR-27b-3p mimic co-overexpression (Fig. 5D).

### FBLN5 silence reverses the LINC01089 effect in thyroid cancer

**Fig. 6:**
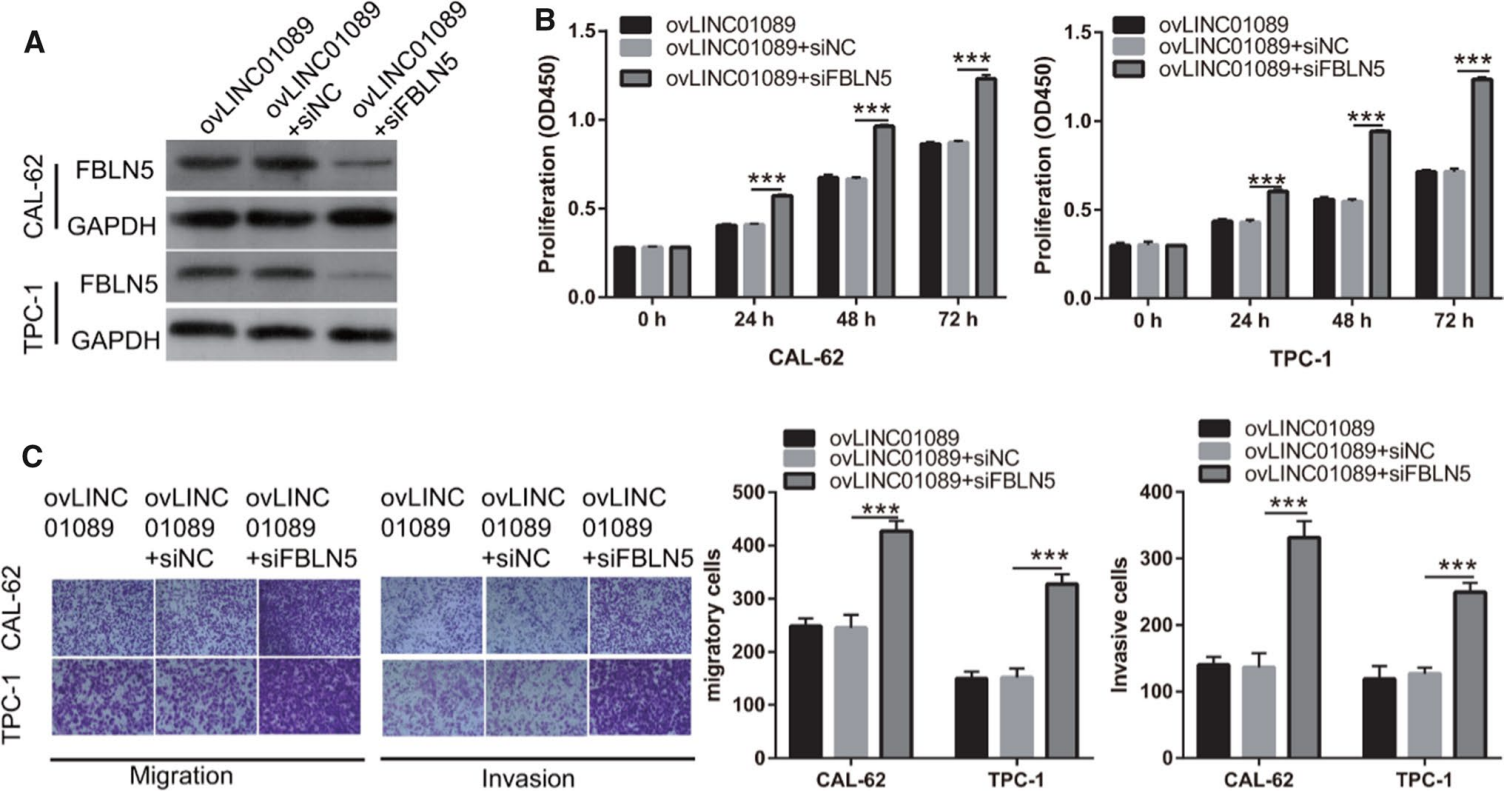
FBLN5 silence promoted the malignant progression potential of ovLINC01089-transfected thyroid cancer cells. **A** FBLN5 protein in TPC-1 and CAL-62 cells was measured via western blotting after co-transfection of ovLINC01089 and siFBLN5. **B** Proliferation was evaluated by the CCK-8 assay after co-transfection. **C** Migration and invasion were assessed by the Transwell assay after co-transfection (magnification, 100×). Histograms represent the mean ± SD (n = 3). ***p < 0.001

Next, we investigated whether FBLN5 is involved in the influence of LINC01089 on the biological function of thyroid cancer. We performed rescue experiments and co-transfected si FBLN5 (siNC) and ovLINC01089 into TPC-1 and CAL-62 cells. Compared with that in the ovLINC01089 + siNC group, the FBLN5 protein level in the ovLINC01089 + siFBLN5 group was significantly down-regulated in both TPC-1 and CAL-62 cells (Fig. 6A). Meanwhile, the proliferation, migration, and invasion capacity of thyroid cancer cells were increased in the ovLINC01089 + siFBLN5 group (Fig. 6B and C). This result demonstrated that FBLN5 knockdown promotes the progression potential of thyroid cancer and weakens the LINC01089 effect.

## Discussion

Neck lymph nodes and distant metastasis in thyroid cancer promote tumor recurrence and a low survival rate [3–6]. In this study, LINC01089 expression in thyroid cancer samples and cells was markedly reduced. Lower LINC01089 expression was significantly associated with higher T/N stages. These results verified that LINC01089 might be involved in the development of thyroid cancer. LINC01089 acts as a tumor suppressor that inhibits malignant progression capacity in breast cancer, NSCLC, and colorectal cancers [11–13]. Similarly, our study found that LINC01089 overexpression blocked the malignant progression capacity of thyroid cancer, verifying that LINC01089 also acts as a tumor suppressor.

Additionally, we found that miR-27b-3p expression was enhanced in thyroid cancer tissues and cells. It is controversial whether abnormally expressed miR-27b-3p acts as an oncogene or an anti-oncogene in cancer. miR-27b-3p inhibited malignant progression capacity by targeting RUNX1, Nrf2, and MARCH7 in gastric cancer, esophageal squamous cell carcinoma, and endometrial cancer, respectively [25–27]. In contrast, miR-27b-3p accelerated tumor progression via PPARG and HOXA10 in triple-negative breast cancer and colorectal cancer, respectively [28, 29]. In addition, the knockdown of miR-27b-3p reduced the chemoresistance in doxorubicin-resistant thyroid cancer cells [30], showing that miR-27b-3p plays an oncogene role in thyroid cancer. Consistently, we found that miR-27b-3p plays an oncogene role in thyroid cancer. Additionally, LINC01089 can bind with miR-27b-3p and inhibit its expression. miR-27b-3p overexpression successfully weakened the LINC01089 effect in thyroid cancer. These results suggest that LINC01089 blocks the malignant progression of thyroid cancer cells by sponging and inhibiting miR-27b-3p.

Subsequently, miRNAs bind with the 3ʹ-UTR of the target gene to reduce its protein level, thereby exerting a regulatory function. FBLN5 was discovered in this study to be the targeted gene of miR-27b-3p in thyroid cancer. In addition, FBLN5 expression was reduced by miR-27b-3p overexpression, suggesting that FBLN5 is the downstream target gene of LINC00987 in thyroid cancer. FBLN5 expression was decreased in lung adenocarcinoma, cervical cancer, and prostate cancer [21, 31, 32]. FBLN5 overexpression reduces tumor angiogenesis, suppresses lung adenocarcinoma and ovarian cancer progression, and has anti-tumor function [21, 33, 34]. In addition, FBLN5 acted as a target gene that can be regulated by miRNAs, such as miR-552/370/27a-3pp, thereby exerting the function of an anti-oncogene in NSCLC, breast cancer, and ovarian carcinoma [22, 35, 36]. Here, FBLN5 was found to be underexpressed in thyroid cancer, and its expression was enhanced by LINC00987 overexpression. Furthermore, the knockdown of FBLN5 promoted the malignant progression of thyroid cancer cells, reversing the effect of LINC00987. These results suggested that FBLN5 acts as a tumor suppressor and was the downstream target gene of LINC00987 in thyroid cancer. LINC01089 exerts a tumor suppressor effect by up-regulating the level of FBLN5. Combined with the regulatory relationship between miR-27b-3 and FBLN5 and the adsorption relationship between LINC01089 and miR-27b-3p, these results suggested that LINC01089 upregulates the expression of FBLN5 by adsorbing miR-27b-3p and exerts a tumor suppressor function in thyroid cancer.

However, this study had some limitations. First, the other target genes of miR-27b-3p need to be further explored. In addition, the function of LINC01089 in thyroid cancer needs to be further verified at the animal level. Finally, because of the small number of thyroid cancer patients that experience metastases and death, the relationship between LINC01089 expression and recurrence and death needs to be further investigated.

In conclusion, LINC01089 plays a tumor-suppressive role in malignant progression by inhibiting miR-27b-3p and increasing FBLN5 protein, confirming that LINC01089 has tremendous potential for use in the treatment of thyroid cancer.

## Data Availability

All data acquired during the study appear in the submitted article.

## Abbreviations

FBLN5: Fibulin-5
LncRNA: Long noncoding RNAs
NSCLC: Non-small cell lung cancer
AGO2: Argonaute2
RIP: RNA immunoprecipitation
RT-qPCR: Reverse-transcription quantitative polymerase chain reaction
ANOVA: One-way analysis of variance
SD: Standard deviation
T: Tumor size
N: The metastasis of regional lymphnodes
M: Distant metastasis
OS: Overall survival
PFI: Progression-free interval
